# Maternal serum levels of perfluoroalkyl substances in early pregnancy and offspring birth weight

**DOI:** 10.1038/s41390-019-0720-1

**Published:** 2019-12-13

**Authors:** Sverre Wikström, Ping-I Lin, Christian H. Lindh, Huan Shu, Carl-Gustaf Bornehag

**Affiliations:** 1 School of Medical Sciences, Örebro University, Örebro, Sweden; 2 Department of Health Sciences, Karlstad University, Karlstad, Sweden; 3 Division of Occupational and Environmental Medicine, Lund University, Lund, Sweden; 4 Department of Environmental Science and Analytical Chemistry, Stockholm University, Stockholm, Sweden; 5 Department of Preventive Medicine, Icahn School of Medicine at Mount Sinai, New York City, NY USA

## Abstract

**Background:**

Perfluoroalkyl substances (PFASs) are widespread, bioaccumulating, and persistent and show placental transfer. Emerging research indicates associations between prenatal exposure and low birth weight. The aim of this study was to assess the associations between first trimester exposure to PFASs and birth weight (BW) in the Swedish Environmental, Longitudinal, Mother and child, Asthma and allergy (SELMA) study and examine whether associations differ between girls and boys.

**Methods:**

Eight PFASs were analyzed in maternal serum (median: 10 weeks of pregnancy). Associations between prenatal PFAS exposure and birth outcomes with BW, BW for gestational age, and birth small for gestational age (SGA) were assessed in 1533 infants, adjusted for potential confounders and stratified by sex.

**Results:**

Increased maternal perfluorooctane sulfonate (PFOS), perfluorooctanoic acid (PFOA), perfluorononanoic acid (PFNA), perfluorodecanoic acid (PFDA), and perfluoroundecanoic acid (PFUnDA) were associated with lower BW, lower BW for gestational age, and SGA birth. Associations were significant only in girls, where prenatal exposure in the upper quartile was associated with a 93–142-g lower BW when compared with that of the lowest quartile exposure. The associations were not mediated by effects on gestational age.

**Conclusions:**

We found associations between prenatal exposure for five different PFASs and birth weight, with more pronounced associations in girls than in boys.

## Introduction

Perfluoroalkyl substances (PFASs) are organic compounds with stain, grease, and water-repellant properties that are valuable in textiles, pans, and food packaging, such as grease-proof paper. Several PFASs are bioaccumulating,^1^ and they are extremely persistent in the environment.^1^ The half-lives of PFAS compounds have been estimated to be in the range of 3–5 years,^2^ and some of them have been found to be transferable through the placenta.^3^ Therefore, human exposure, which is shown worldwide, will continue for a long time, despite any regulations or phasing-out.

Several studies over the past decade have shown that higher prenatal exposure to perfluorooctane sulfonate (PFOS) and perfluorooctanoic acid (PFOA) could predict a lower birth weight (BW). In a systematic review of 18 studies (2014), Johnson et al.^4^ concluded that there is “sufficient” human evidence that prenatal exposure to PFOA reduces fetal growth. Prenatal exposure was in most cases determined through PFOA analyses in maternal serum/plasma at highly different pregnancy lengths or in cord plasma. In another systematic review of 14 studies (2015), Bach et al.^5^ reported that higher maternal PFOS and PFOA levels were associated with decreased BW in most studies, but only some results were significant. On the other hand, Steenland et al. presented very recently an updated meta-analysis providing only modest support for decreased BW with increasing PFOA. Serious concerns were raised about reverse causality in studies utilizing late pregnancy sampling.^6^ Consequently, the magnitude and relevance of associations with BW have remained unclear^5^ and exposure measurements in early pregnancy are crucial to limit the potential of confounding. Even more important, however, is that PFASs also include many other compounds more recently introduced, for which community exposure is not declining as it is for PFOS and PFOA.^7^ There has been very limited research on the link between these PFAS compounds and BW, and most previous studies were small. Many of the published findings seem to be inconsistent, partly due to the variable times of collecting the biospecimen for measurements (e.g., late trimester during the pregnancy or by delivery).

Some investigations have shown sex-related differences in the association between PFASs and BW, but the results are strikingly inconsistent between different populations. Having the proposed pathways for the PFAS influence on BW in mind, including sex hormone signaling,^8–10^ such sexual dimorphism must be considered. However, investigations of sex differences have not been extensively explored in most studies. As part of the assessments of the mechanisms behind the effects of PFASs on BW, epidemiological associations of lower BW primarily via shorter pregnancies must also be ruled out.^11^

The aim of the present study was therefore to evaluate the association between early pregnancy exposure to eight PFAS compounds and BW in the Swedish Environmental Longitudinal, Mother and child, Asthma and allergy (SELMA) study, specifically focusing on differences according to the sex of the child.

## Methods

### Study population

SELMA is a longitudinal pregnancy cohort study designed to investigate the impacts of early life exposure to environmental factors on growth, development, and chronic diseases in children. In the full cohort, blood serum samples were obtained from 2355 pregnant women in weeks 3–27 of pregnancy at their first visit at their antenatal care center in Värmland between September 2007 and March 2010, a period of approximately 2.5 years.^12^ In the present study group, the median gestation at sampling was 10 weeks, where 96% of the samples were collected during the first trimester and the remaining during the first weeks thereafter.

Children born by the participating women and for which outcome data, exposure data, and all statistical covariates (as presented below) were available constituted the present study group. In order to avoid dependent data, all twins (*n* = 32) were excluded. In total, the study group included *N* = 1533 infants. Data were retrieved from the Swedish Medical Birth Register, where Swedish births are reported by healthcare professionals after delivery regarding infant BW and gestational age (GA), as well as maternal weight, age, and parity.

### PFASs and cotinine in prenatal serum

Serum was analyzed by liquid chromatography tandem mass spectrometry at The Department of Occupational and Environmental Medicine in Lund, Sweden. A detailed description of the method was previously presented by Lindh et al.^13^ Briefly, aliquots of 100 μL of serum were added with labeled internal standard for all compounds and precipitated using acetonitrile. Eight PFASs were analyzed, including PFOS, PFOA, perfluorohexane sulfonate (PFHxS), perfluorononanoic acid (PFNA), perfluorodecanoic acid (PFDA), perfluoroundecanoic acid (PFUnDA), perfluoroheptanoic acid (PFHpA), and perfluorododecanoic acid (PFDoDA). Compounds with <50% of samples above the limit of detection (LOD) were excluded from the analyses. All values below the LOD were set to half the LOD.

In the same samples, cotinine was analyzed and used as a marker for smoking. For descriptive purposes, women were categorized as nonsmokers if their cotinine levels were below the LOD at 0.2 ng/mL, as active smokers if their cotinine levels were >15 ng/mL, and passive smokers if their levels were in between.^14^ The laboratory is part of a quality control program between analytical laboratories for analyses of PFOS and PFOA, coordinated by Professor Hans Drexler, Institute and Outpatient Clinic for Occupational, Social and Environmental Medicine, University of Erlangen-Nuremberg, Germany.

### Outcome measures

BW (g) was used as a continuous outcome variable but was also expressed as sex- and GA-specific *z*-score (i.e., BW for GA, hereafter “BW-SDS”) according to a national growth reference extending into early preterm ages.^15^ Infants with BW below the 10th percentile for GA and sex were defined as small for gestational age (SGA).

### Statistical analysis

We performed the analyses using a compound-by-compound approach to investigate the individual PFASs in maternal serum as predictors of BW and BW-SDS using multiple linear regression models and as predictors of being born SGA (multiple logistic regression models).

Maternal serum PFASs and cotinine concentrations were transformed with the natural logarithm to achieve approximate normal distributions. For comparisons between compounds, irrespective of the serum level range and for identification of effects limited to parts of the exposure range, modeling was thereafter also performed per quartile range of the PFAS exposure, with the lowest quartile used as a reference and with the same adjustments as for BW (presented below).

In stepwise regression models, the following determinants of BW and potential confounders, as based on previous literature,^4,5,16^ were explored as covariates: sex and GA (days according to ultrasonography assessment) of the child, maternal weight (kg), age (years), parity (I-para, II-para, and III-para+), cotinine exposure (ln cotinine), and education level (university degree vs lower), as well as pregnancy week of serum sampling and fish intake in the family during pregnancy. Fish intake was assessed using an index created based on the sum of all fish and seafood intake frequencies, according to food questionnaires.^17^ In the stepwise procedure, variables that according to changes in the *R*^2^ statistics improved regression models were included as covariates in the final modeling of serum PFAS levels as predictors of BW. Based on the stepwise regression, a uniform model for both sexes and for all PFASs was considered suitable and applied, including sex, GA, parity, maternal weight, and cotinine concentration (for the linear models of BW).

In linear models of BW-SDS and in the logistic regression models of being born SGA, the same covariates were used except for GA (already considered within these outcome variables). Fish intake, pregnancy week of serum sampling, maternal age, and education level did not contribute to models according to the stepwise modeling and were hence not included. Multicollinearity analysis was assessed for the models by calculating the variance inflation factor (VIF) and indicated, as previously shown in univariate analyses within the SELMA study,^17^ that fish intake may be a major source of PFAS exposure (i.e., constituting a proxy for exposure in the present dataset), making it unsuitable as a covariate in models of infant BW. For the final models presented here, VIF was <1.2 for all models, and we did not regard regression estimates to be biased by multicollinearity. The final models were also reanalyzed restricted to pregnancies where maternal serum was collected during the first trimester.

To test the hypothesis of the sex-specific effects in the association between PFAS exposure and BW, we stratified all analyses by sex and applied the same multiple linear regression models in each group. To determine whether sex-specific associations between PFAS exposure and BW could be statistically significant, we evaluated whether PFAS exposure interacted with sex to jointly influence BW in the full sample. An interaction term between sex and exposure was included as a predictor variable together with the covariates. Here, ln-PFAS levels were centered by subtracting the mean to minimize multicollinearity. Improvement in the *R*^2^ statistic together with a statistically significant interaction term was considered evidence for modification by sex.

Note that GA may constitute an intermediate along a pathway from PFAS exposure to lower BW. Therefore, all associations were evaluated here by adjustments for GA but also utilizing BW-SDS (i.e., BW for GA) and SGA birth as separate outcome measures and by a statistical mediator analysis^18^ on this association. Regression models of PFASs as a predictor of BW were also rerun after exclusion of preterm (<37 weeks) born infants (*n* = 56), as well as without adjustment for GA.

All statistical analyses were performed using IBM SPSS Statistics for Windows, Version 22.0. (NY: IBM Corp, USA).

The Regional Ethical Review Board in Uppsala, Sweden approved the study protocol, and written consent was obtained from each participating woman.

## Results

### Study population

Complete outcome data, exposure data, and all statistical covariates as presented above were available for 1533 mother–infant pairs, hence constituting the study group, presented in Table 1. The median (interquartile range (IQR)) BW for girls was 3565 (3210–3905) and 3670 (3316–4060) g for boys (*p* < 0.001 for the difference between sexes), while there was no difference between girls and boys regarding BW-SDS (−0.048 (−0.686 to 0.642) SDS and −0.171 (−0.793 to 0.657) SDS, respectively (*p* = 0.12)). There were no other sex differences for the variables in Table 1 (data separated per sex not shown). For eligible children born within the SELMA study but excluded owing to missing covariate data (*n* = 122), there were no differences in maternal PFAS or cotinine concentrations, parity, age, education, or GA compared with the present study group.

**Table 1:** Descriptive data on the 1533 children constituting the study population.

Girls/boys; *N* (%)	732 (47.7%)/801 (52.3%)
Birth weight; g (IQR)	3630 (3290–3998)
Birth weight *z*-score; SDS (IQR)	−0.09 (−0.71–0.65)
Small for gestational age^a^; *N* (%)	168 (11%)
Gestational age; days (IQR)	281 (274–287)
Preterm birth^b^; *N* (%)	56 (3.7%)
Maternal age; years (IQR)	31 (28–34)
Maternal weight at enrollment; kg (IQR)	67 (60–75)
Maternal parity; *N* (%)	
I-para	704 (45.9%)
II-para	559 (36.5%)
III-para+	270 (17.6%)
Maternal smoking status^c^; *N* (%)	
Non-smoker	1345 (87.7%)
Passive smoker	82 (5.3%)
Active smoker	106 (6.9%)

^a^Small for gestational age is defined as birth weight below the 10th percentile for GA and sex

^b^Preterm birth is defined as gestational age <37 weeks

^c^Smoke exposure assessed according to serum cotinine levels at enrollment (“active smoker” if cotinine levels >15 ng/mL; “passive smoker” if cotinine level 0.2–15 ng/mL)

### Prenatal PFAS exposure

Among the eight studied compounds, PFOS had the highest concentrations, followed by PFOA, as described in Table 2. Owing to the large number (>50%) of samples with levels below the LOD, we excluded PFDoDA data from further analysis. There were no differences in PFAS serum levels between pregnancies with male or female fetuses (data not shown).

**Table 2:** Prenatal exposure to eight PFAS, measured as maternal serum concentrations (ng/mL) during early pregnancy.

Compound	Geometric mean [95% CI]	Median (IQR)	95th %	LOD	Above LOD (%)
PFOS	5.35 [5.21–5.50]	5.38 (3.97–7.60)	10.34	0.06	100
PFOA	1.60 [1.56–1.65]	1.61 (1.11–2.30)	3.18	0.02	100
PFHxS	1.31 [1.28–1.35]	1.23 (0.86–1.99)	2.94	0.03	100
PFNA	0.54 [0.53–0.56]	0.53 (0.39–0.73)	1.02	0.01	100
PFDA	0.26 [0.26–0.27]	0.26 (0.19–0.34)	0.50	0.02	100
PFUnDA	0.21 [0.21–0.22]	0.23 (0.15–0.33)	0.44	0.02	99.5
PFHpA	0.018 [0.017–0.019]	0.02 (<LOD–0.04)	0.077	0.01	73.9
PFDoDA	0.027 [0.026–0.027]	<LOD (<LOD–0.05)	0.08	0.03	46.7

*LOD* limit of detection

### Prenatal PFAS exposure and BW outcomes

In multiple regression models adjusted for sex, GA, maternal weight, parity, and cotinine concentration, increased maternal serum concentrations of five out of seven assessed PFAS compounds (PFOS, PFOA, PFNA, PFDA, and PFUnDA) were significantly associated with lower BW and with lower BW-SDS (Table 3 for BW and Supplementary Table 4 for BW-SDS).

**Table 3:** Associations between prenatal PFAS exposure^a^ and birth weight^b^, together with odds ratios for birth weight small for gestational age in 1533 children.

	All children		Girls		Boys	
	BW (g)	SGA	BW (g)	SGA	BW (g)	SGA
	*β* (95% CI)	OR (95% CI)	*β* (95% CI)	OR (95% CI)	*β* (95% CI)	OR (95% CI)
PFOS						
Per ln-unit	−46 (−88; −3)	1.19 (0.87; 1.64)	−85 (−145; −25)	1.40 (0.83; 2.35)	−13 (−73; 47)	1.08 (0.72; 1.63)
Q1	Reference	Reference	Reference	Reference	Reference	Reference
Q2	−27 (−89; 35)	0.69 (0.43; 1.08)	−32 (−115; 52)	0.89 (0.39; 2.03)	−28 (−118; 63)	1.26 (0.67; 2.37)
Q3	−22 (−84; 41)	0.79 (0.53; 1.18)	−51 (−137; 34)	0.82 (0.36; 2.03)	5 (−86; 96)	0.86 (0.45; 1.67)
Q4	−80 (−144; −16)	1.56 (1.09; 2.22)	−142 (−231; −54)	2.05 (1.00; 4.21)	−28 (−119; 63)	1.30 (0.70; 2.40)
PFOA						
Per ln-unit	−68 (−112; −24)	1.43 (1.03, 1.99)	−86 (−145; −26)	1.96 (1.18; 3.28)	−49 (−113; 15)	1.16 (0.75; 1.78)
Q1	Reference	Reference	Reference	Reference	Reference	Reference
Q2	27 (−35; 89)	0.77 (0.45; 1.32)	30 (−55; 115)	1.00 (0.40; 2.51)	26 (−66; 116)	0.67 (0.34; 1.31)
Q3	−41 (−106; 23)	0.96 (0.57; 1.61)	−36 (−124; 52)	1.64 (0.71; 3.83)	−44 (−139; 50)	0.66 (0.33; 1.29)
Q4	−90 (−159; −91)	1.44 (0.86; 2.40)	−136 (−231;−40)	2.33 (1.00; 5.43)	−47 (−147; 54)	1.04 (0.54; 2.01)
PFHxS						
Per ln-unit	−0.1 (−38; 38)	0.96 (0.72; 1.27)	−14 (−68; 39)	1.14 (0.73; 1.80)	−13 (−67; 41)	0.84 (0.58; 1.22)
Q1	Reference	Reference	Reference	Reference	Reference	Reference
Q2	−4 (−66; 58)	1.37 (0.86; 2.20)	30 (−56; 116)	1.77 (0.78; 3.99)	−39 (−129; 50)	1.24 (0.69; 2.23)
Q3	−15 (−78; 48)	0.89 (0.54; 1.47)	28 (−59; 115)	1.05 (0.44; 2.49)	−51 (−141; 39)	0.82 (0.44; 1.54)
Q4	−6 (−69; 57)	1.04 (0.63; 1.69)	−16 (−104; 71)	1.76 (0.79; 3.90)	1 (−90; 92)	0.73 (0.38; 1.41)
PFNA						
Per ln-unit	−46 (−89; −4)	1.38 (1.02, 1.87)	−52 (−117; −2)	1.34 (0.85; 2.11)	−50 (−113; 14)	1.42 (0.94; 2.17)
Q1	Reference	Reference	Reference	Reference	Reference	Reference
Q2	7 (−55; 69)	0.83 (0.49; 1.38)	−2 (−86; 82)	0.66 (0.29; 1.52)	15 (76; 106)	0.97 (0.50; 1.89)
Q3	−39 (−102; 24)	1.14 (0.70; 1.85)	−49 (−137; 38)	1.39 (0.66; 2.90)	−28 (−119; 64)	0.95 (0.50; 1.82)
Q4	−33 (−96; 31)	1.23 (0.77; 1.99)	−66 (−153; 20)	1.22 (0.59; 2.53)	1 (−94; 95)	1.24 (0.66; 2.33)
PFDA						
Per ln-unit	−58 (−103; −13)	1.46 (1.06, 2.01)	−69 (−133; −6)	1.62 (0.98, 2.67)	−47 (−112;17)	1.36 (0.90; 2.07)
Q1	Reference	Reference	Reference	Reference	Reference	Reference
Q2	−23 (−85; 39)	1.03 (0.62; 1.69)	−42 (−126; 42)	0.86 (0.37; 2.00)	−8 (−99; 82)	1.18 (0.63; 2.23)
Q3	−39 (−101; 23)	1.07 (0.65; 1.76)	−74 (−160; 13)	1.20 (0.54; 2.67)	−8 (−98; 81)	0.99 (0.52; 1.89)
Q4	−69 (−132; −5)	1.50 (0.94; 2.38)	−116 (−204; −27)	1.95 (0.94; 4.06)	−27 (−118; 64)	1.21 (0.66; 2.23)
PFUnDA						
Per ln-unit	−13 (−49; 22)	1.21 (0.92; 1.58)	−24 (−75; 27)	1.08 (0.70; 1.67)	−6 (−55; 42)	1.29 (0.82; 1.83)
Q1	Reference	Reference	Reference	Reference	Reference	Reference
Q2	−67 (−153; 19)	1.24 (0.77; 2.01)	−67 (−153;19)	2.09 (0.94; 4.63)	41 (−48; 130)	0.90 (0.48; 1.68)
Q3	−42 (−128; 44)	0.85 (0.51; 1.43)	−42 (−128; 44)	1.01 (0.42; 2.44)	46 (−44; 136)	0.81 (0.43; 1.56)
Q4	−46 (−110; 17)	1.52 (0.95; 2.44)	−93 (−183; −3)	1.92 (0.86; 4.25)	−10 (−100; 80)	1.36 (0.76; 2.46)
PFHpA						
Per ln-unit	−1 (−24; 21)	1.06 (0.90; 1.25)	−4 (−34; 26)	1.06 (0.83, 1.36)	−0.03 (−33; 32)	1.07 (0.86; 1.37)
Q1	Reference	Reference	Reference	Reference	Reference	Reference
Q2	−0 (−62; 61)	0.92 (0.57; 1.48)	17 (−70; 103)	0.92 (0.44; 1.92)	−15 (−102; 73)	0.94 (0.50; 1.75)
Q3	3 (−59; 65)	0.78 (0.48; 1.27)	−21 (−106; 65)	0.75 (0.35; 1.65)	23 (−65; 112)	0.78 (0.42; 1.45)
Q4	31 (−31; 93)	1.25 (0.85; 1.84)	27 (−57; 112)	1.31 (0.66; 2.60)	33 (−58; 124)	1.15 (0.65; 2.04)

All analyses were adjusted for maternal weight, parity (three categories) and cotinine levels. Analyses including both boys and girls were in addition adjusted for sex and analyses of BW were adjusted for GA. SGA was defined as BW  10th percentile for sex and GA

^a^Associations with PFAS are presented per ln-unit and by quartiles of exposure, as related to

^b^Birth weight (g) and odds ratios (adjusted) for birth weight small for gestational age

In the full sample including both girls and boys, a ln-unit increase in prenatal exposure to PFOS, PFOA, PFNA, and PFDA (all closely corresponding to an increase from the 25th to the 75th percentile) was associated with a decrease in BW in the range of 46–68 g or 0.10–0.15 SDS. Children in the upper quartile of prenatal exposure for PFOS, PFOA, and PFDA were 69–90 g lighter than children born in the lower quartile of prenatal exposure (Table 3).

Prenatal PFAS exposure for PFOS, PFOA, PFNA, and PFDA was also significantly associated with being born SGA when adjusted for potential confounders (Table 3).

For comparison, smoke exposure (active or passive during pregnancy according to cotinine analyses) in all models was related to 96–102 g lower BW, with −96 (95% confidence interval (CI): −162; −30) and −94 (95% CI: −160; −27) g in the models including PFOS and PFOA (regression coefficients for smoke exposure in other PFAS models are not shown).

Although the pregnancy week of serum sampling was not identified as a relevant predictor variable in the stepwise regression, all analyses were rerun restricted to maternal serum samples collected during the first trimester. In this sensitivity analysis, there were practically no changes at all in regression coefficients (data not shown).

### Stronger associations for girls

**Fig. 1:**
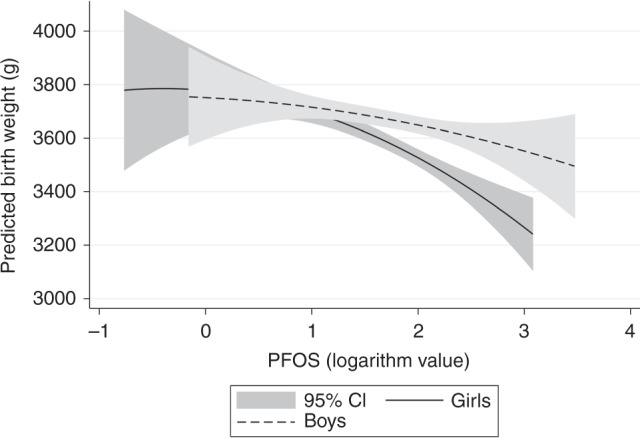
Relationship between predicted birth weight (g) and prenatal PFOS exposure. Analyses of the predicted birth weight were stratified by sex and adjusted for gestational age, maternal weight, parity, and cotinine level.

According to analyses stratified by sex, the associations between prenatal PFAS exposure and BW were stronger in girls than in boys (Table 3 and Fig. 1, as well as Supplementary Table 4) and significant in girls only in all cases (PFOS, PFOA, PFNA, PFDA, and PFUnDA). *p* Values for the interaction term between PFASs and sex did not support a statistically significant interaction effect (*p* = 0.06 for PFOS, *p* = 0.07 for PFOA, *p* = 0.25 for PFNA, *p* = 0.33 for PFDA, *p* = 0.77 for PFUnDA, *p* = 0.62 for PFHxS, and *p* = 0.83 for PFHpA).

### Mediation by GA

The analyses of GA as a potential mediator of the association between prenatal PFAS exposure and BW showed no such effect. First, PFAS levels were not associated with GA in bivariate analyses. Second, increasing PFAS levels were (as presented above) associated both with lower BW in models adjusting for GA and with lower BW for GA (BW-SDS) according to Supplementary Table 4. Third, the statistical mediation analysis indicated no mediation by GA. Finally, the associations between prenatal exposure and BW remained unchanged when preterm-born infants (*n* = 56) were excluded, and there were no significant or consistent changes in associations with PFASs if GA was excluded from the statistical covariates (data not shown).

## Discussion

### Prenatal PFAS exposure and BW outcomes

The present study shows significant associations between prenatal exposure to five different perfluoroalkyl acids (PFOS, PFOA, PFNA, PFDA, and PFUnDA) and low BW when adjusted for potential confounders (Table 3). The present results were highly consistent between different BW outcome measures since we also show associations with lower BW-SDS (i.e., BW for GA) and increased risk of being born SGA (according to sex- and nation-specific reference data) with increased exposure.

Previously, two systematic reviews including 18 previous studies (with a meta-analysis of 9)^4^ and 14 studies, respectively,^5^ concluded that higher prenatal exposure to PFOA is associated with decreased BW. For PFOS, only a limited number of the reviewed studies showed significant associations.^5^ Although the serum levels identified in our cohort are comparably low, these exposure levels of PFOA and PFOS seem to contribute significantly to BW reduction. Owing to the environmental persistence, we conclude that this will be the case for years to come.

In the present study, we also found significant associations between BW and three additional PFASs (PFNA, PFDA, and PFUnDA). Exposure to these three compounds is substantial,^16,19,20^ but they are still much less studied compared with PFOA and PFOS, and previous studies were, with a few exceptions,^11,19,21^ small sized. The findings have been inconsistent,^19–23^ with indications of slightly lower BW after higher prenatal exposures in only a few studies.^20,22–24^ Furthermore, other investigations on the newer and more long-chained PFASs show considerable differences in the timing of sampling (during pregnancy, at delivery, or thereafter). This makes comparisons cumbersome but stresses the importance of the present contribution. Among PFASs not related to BW in the present study, it should be noted that, for PFHpA, 26% of samples fell below the LOD and were therefore set to half of the LOD in statistical analyses. This is unlikely to have any major effect on analysis per quartile but may limit the use of PFHpA as a linear variable.

### Different associations in girls and boys

In analyses stratified by sex, the associations between prenatal PFAS exposure and BW were significant for girls only in all cases (PFOS, PFOA, PFNA, PFDA, PFUnDA). This was irrespective of whether BW or the sex-specific *z*-score (BW-SDS and analyses of SGA birth) was assessed. In addition, regression coefficient estimates pointed toward more pronounced associations in girls compared with boys. Analyses per quartile of PFAS exposure indicated that the differences between girls and boys were largest in the upper exposure range (Table 3 and Fig. 1). Although this suggests important sex-specific associations between prenatal PFAS exposure and BW, testing of it with sex interaction analyses as performed here failed to show statistical significance (*p* = 0.06 for PFOS and *p* = 0.07 for PFOA).

The findings of a greater negative impact of PFASs on BW in girls compared with boys are in line with some previous findings predominantly on PFOS,^20,25,26^ but this has not been confirmed in all previous cohorts^19,23^ and even contradicts results from another Scandinavian population by Lauritzen et al.^27^ There, associations between prenatal PFOA and PFOS and lower BW and higher odds for SGA birth were found in a Swedish (*n* = 159) subgroup (but not Norwegian, *n* = 265) of infants and only in boys (*n* = 81). The reasons for the contrary findings compared to the present study may originate in the considerably different study designs: one-third of infants were born SGA in the case–control study by Lauritzen, and in turn, 83% of the Swedish infants came from an already identified SGA high-risk group of already parous mothers (blood sampled at mid pregnancy). Lower plasma expansion—with resulting effects on maternal PFAS levels during pregnancy—is closely associated with impaired fetal growth. Since more males than females in the small study sample of Lauritzen et al. were SGA, this may explain some of the sex differences compared to our study. In our study, approximately 12% of infants were SGA, and there were no differences between boys and girls for exposure levels or background variables (Table 1, data separated by sex not shown), other than the expected difference in absolute BW (but not BW-SDS).

The mechanisms behind the influence of PFASs on fetal growth are largely unknown, and hence, the same applies to the suggested sex differences. Emerging research has shown that PFASs may influence human sex hormone biosynthesis, serum levels, and receptor function,^8–10,28^ providing possible explanations for the sexual dimorphism in the human biological response to exposure. However, these mechanisms may also be questioned based on other recent in vitro findings.^29^ Interestingly, the present sex-specific results are in line with associations between prenatal PFAS exposure and metabolic outcomes such as BMI development later in life.^30,31^

### Timing of prenatal exposure measurements

Sampling for chemical analyses early in pregnancy may be important not only to establish an early developmental effect of PFAS exposure but also to avoid the potential of confounding due to pregnancy-related changes in the glomerular filtration rate and hemodynamics.^3,32^ A recent meta-analysis by Steenland et al. suggests that the timing of exposure measurement is critically important for the association between maternal PFOA serum concentrations and offspring BW.^6^ Sampling later in pregnancy may be related to more problems with confounding or reverse causality issues.^6^ Thanks to the prospective design, blood samples in the present study were drawn very early (at a median of 10 weeks of gestation), with 96% during the first trimester and the remaining during the early weeks of the second trimester. Here we found no evidence of an impact of gestational week of sampling when this was tested as a covariate of multiple regression models, and analyses restricted to first trimester samples were no different from the overall findings above.

Other studies on the relationship between PFOA and PFOS and BW have utilized early pregnancy sampling with community exposure level ranges similar to ours.^19,21,33,34^ For PFOA, our findings are in line with most previous data^4,5,33,34^ based on similar GA for sampling, although effect sizes in terms of regression coefficients were generally larger in our cohort (which is also among the largest reported). For PFOS, the picture is much less clear, although at least some comparable studies report an association between early pregnancy PFOS exposure and lower BW.^19,21,34^

### Public health relevance

In the present study, prenatal exposure to PFOA within the fourth quartile compared with first quartile levels was associated with a 136-g lower BW in girls. For comparison, the established effect from maternal smoking in mid-to-late pregnancy is associated with a 170–200-g lower BW.^35^ In the present analyses, tobacco smoke exposure (active or passive smoking according to cotinine analyses) during early pregnancy was in all models related to approximately a 100-g lower BW.

A reduction in BW of this size may have a minor impact on an individual infant. However, a shift in the BW distribution of a population may have consequences from a public health perspective due to increased proportions of infants with low BW or born SGA. From this point of view, we also regard the present odds ratios for PFASs as predictors of SGA birth to have clinical implications. Being born SGA also poses long-term consequences to infants born at term age.^36^ This makes the association between PFAS exposure and this clinical outcome highly worrisome and important for future studies, as already noted in the previous review by Bach et al.^5^ The magnitude of the present associations must hence be considered relevant for public child health.

The continuously building evidence of early developmental effects from PFASs also raises concerns from a lifetime perspective. Lower BW, especially if followed by rapid catch-up growth, is closely related to increased risks of adult obesity, unfavorable metabolic outcomes, and increased risk of cardiovascular disease,^37,38^ consistent with intrauterine programming effects according to the “Developmental Origin of Health and Disease” hypothesis^36,38^ and the originally proposed hypothesis of Barker. Prenatal exposure to several PFASs, but in particular PFOA, has, in several studies, been linked to increases in adiposity measures (e.g., body mass index, skinfolds, and waist measures) during childhood. This has varied between studies with different levels of exposure and conflicts with other studies where null associations were reported.^39,40^

### Methodological considerations

As previously noted,^5^ it is important to rule out that the association between increasing PFAS levels and BW is mediated via shorter GA. Here we performed several different investigations of this issue and found no signs of such indirect effects. Fish intake during pregnancy may also be taken into consideration in assessments of the relationship between PFASs and BW. In several previous studies (although some were conducted before peak community exposures to PFOS and PFOA), fish intake has been related to higher BW. However, fish are also a major source of PFAS exposure in Scandinavia.^41^ In the present study, we included fish intake among the covariates of stepwise models, and maternal fish intake had no statistically significant contribution to BW. The stepwise selection method might still, to some extent, lead to reduced predictive power by discarding variables not correlated with the dependent variable. Furthermore, the number of candidate predictor variables may affect the number of noise variables that enter the model.

Other methodological strengths of the present study, in addition to a variety of measured PFAS compounds and early pregnancy sampling, are the prospective cohort design, including high-quality recording of covariates and a large sample size. Still, residual confounding cannot be excluded. For example, in comparison with some previous studies, our study lacks adjustment for maternal weight gain during pregnancy, although we find little reason to believe it to be covarying with PFAS plasma levels already at 10 weeks of gestation. More importantly, despite samples drawn from 2355 pregnancies originally included in the SELMA study, our study group consisted of *N* = 1533 children. There are several reasons for this, including that serum PFAS analyses were missing, that women had miscarriages or for other reasons left before delivery, that we excluded all multiple births, and finally that statistical covariates were missing for *n* = 122 children. This may to some extent limit the generalizability of the findings, but we find it unlikely to affect the associations within the present study group.

Another limitation is the compound-by-compound approach. Theoretically, a health outcome is simultaneously influenced by multiple environmental factors. Nevertheless, the exposure to several PFASs may be correlated with each other due to common sources. Our findings were consistent across different PFAS compounds, and we regard correction for multiple comparisons overly conservative to be suitable for the investigations on such interrelated compounds. If the single compound’s level could represent the levels of several other compounds, our findings based on single compound analyses may still shed some light on the joint effects of multiple PFAS compounds. However, carefully designed statistical models, such as mixture-based approaches within the PFAS compound class, should be explored in follow-up studies.

## Conclusions

Our data suggested that prenatal exposures to PFOS, PFOA, PFNA, PFDA, and PFUnDA contribute to low BW as well as to being born SGA and that these associations were more pronounced and statistically significant only in girls. These phenomena indicate public health concerns, but more research is warranted to clarify the role of sex and whether the effect persists throughout the entire life course. Owing to the widespread exposure of compounds and their long half-lives in humans, preventive efforts other than phasing out may not be realistic for PFASs with established negative health consequences. Bearing in mind the long-term clinical consequences of being born low weight or SGA, workers in child health should today, however, be aware of the potential negative effects of PFAS developmental exposure at community levels. Based on the indications of sex-specific effects as shown here, it is important not to underestimate the health effects from PFASs in girls, due to the lack of sex-specific investigations. Finally, the findings of associations between more recently introduced PFASs and BW demand more in-depth investigations, which shall take multiple exposures into account.

## Supplementary information

Supplemental Table S1

## Data Availability

Data on chemical exposures (PFASs and cotinine) can be made available to researchers upon request (subject to a review of secrecy). Requests for data should be made to the Head of the Department of Health Sciences, Karlstad University. However, according to the Ethical Review Board decision and obtained personal consent, data collected from clinical care cannot be made freely available. This is because they are subject to secrecy in accordance with the Swedish Public Access to Information and Secrecy Act [OSL 2009:400]. Unique combinations of such data will make a study participant (i.e., patient) identifiable, and consequently, no clinical data will be shared.
