# Supplementary material for: Maternal serum levels of perfluoroalkyl substances in early pregnancy and offspring birth weight
Source: Pediatr Res. 2019 Dec 13;87(6):1093–9. doi: 10.1038/s41390-019-0720-1 (PMC7196936; doi:10.1038/s41390-019-0720-1)
Supplement: Supplementary file 1 — Supplemental Table S1 [file 41390_2019_720_MOESM1_ESM.docx]

**Supplemental Table S1. Associations between prenatal PFAS exposure ^a^ and sex- and gestational age specific Birth Weight Standard Deviation Score (BW-SDS) ^b^ in 1533 children.**

| \|  \| All BW-SDS  β(95% CI) \| Girls BW-SDS  β(95% CI) \| Boys BW-SDS  β(95% CI) \| \| --- \| --- \| --- \| --- \| \| PFOS \|  \|  \|  \| \| Per ln-unit \| -0.100(-0.197; -0.004) \| -0.167(-0.301; -0.034) \| -0.027(-0.166; 0.112) \| \| Q1 \| Reference \| Reference \| Reference \| \| Q2 \| -0.045(-0.185; 0.096) \| -0.044(-0.232; 0.143) \| -0.055(-0.263; 0.153) \| \| Q3 \| -0.024(-0.166; 0.118) \| -0.092(-0.283; 0.100) \| 0.038(-0.171; 0.246) \| \| Q4 \| -0.172(-0.317; -0.027) \| -0.296(-0.494; -0.098) \| -0.066(-0.276; 0.144) \| \| PFOA \|  \|  \|  \| \| Per ln-unit \| -0.152(-0.251; -0.052) \| -0.191(-0.325; -0.057) \| -0.111(-0.258; 0.036) \| \| Q1 \| Reference \| Reference \| Reference \| \| Q2 \| 0.065(-0.076; 0.206) \| 0.065(-0.124; 0.255) \| 0.065(-0.144; 0.274) \| \| Q3 \| -0.088(-0.235; 0.058) \| -0.088(-0.285; 0.109) \| -0.086(-0.302; 0.131) \| \| Q4 \| -0.204(-0.362; -0.047) \| -0.299(-0.513; -0.085) \| -0.117(-0.348; 0.114) \| \| PFHxS \|  \|  \|  \| \| Per ln-unit \| 0.007(-0.079; 0.093) \| 0.031(-0.088; 0.150) \| -0.017(-0.141; 0.107) \| \| Q1 \| Reference \| Reference \| Reference \| \| Q2 \| -0.004(-0.145; 0.138) \| 0.071(-0.122; 0.263) \| -0.081(-0.287; 0.126) \| \| Q3 \| -0.016(-0.158; 0.126) \| 0.062(-0.132; 0.257) \| -0.083(-0.289; 0.123) \| \| Q4 \| -0.008(-0.151; 0.135) \| -0.043(-0.238; 0.153) \| 0.016(-0.194; 0.226) \| \| PFNA \|  \|  \|  \| \| Per ln-unit \| -0.114(-0.211; -0.017) \| -0.114(-0.242; -0.013) \| -0.111(-0.258; -0.036) \| \| Q1 \| Reference \| Reference \| Reference \| \| Q2 \| 0.025(-0.116; 0.165) \| 0.010(-0.177; 0.198) \| 0.034(-0.177; 0.244) \| \| Q3 \| -0.084(-0.228; 0.059) \| -0.113(-0.309; 0.083) \| -0.056(-0.266; 0.155) \| \| Q4 \| -0.081(-0.226; 0.064) \| -0.145(-0.339; 0.049) \| -0.019(-0.235; 0.197) \| \| PFDA \|  \|  \|  \| \| Per ln-unit \| -0.147(-0.250; -0.044) \| -0.163(-0.305; -0.022) \| -0.132(-0.281; 0.017) \| \| Q1 \| Reference \| Reference \| Reference \| \| Q2 \| -0.077(-0.218; 0.063) \| -0.096(-0.284; 0.092) \| -0.068(-0.277; 0.141) \| \| Q3 \| -0.085(-0.227; 0.056) \| -0.167(-0.360; 0.026) \| -0.015(-0.221; 0.191) \| \| Q4 \| -0.179(-0.323; -0.035) \| -0.266(-0.464; -0.068) \| -0.101(-0.310; 0.108) \| \| PFUnDA \|  \|  \|  \| \| Per ln-unit \| -0.051(-0.131; 0.029) \| -0.064(-0.179; 0.051) \| -0.044(-0.156; 0.068) \| \| Q1 \| Reference \| Reference \| Reference \| \| Q2 \| -0.038(-0.179; 0.102) \| -0.165(-0.357; 0.027) \| 0.066(-0.139; 0.271) \| \| Q3 \| -0.003(-0.145; 0.138) \| -0.099(-0.291; 0.093) \| 0.074(-0.132; 0.281) \| \| Q4 \| -0.138(-0.283; 0.006) \| -0.225(-0.425; -0.025) \| -0.072(-0.279; 0.136) \| \| PFHpA \|  \|  \|  \| \| Per ln-unit \| -0.006(-0.056; 0.045) \| -0.008(-0.076; 0.060) \| -0.005(-0.081; 0.070) \| \| T1 \| Reference \| Reference \| Reference \| \| T2 \| 0.025(-0.097; 0.147) \| 0.073(-0.091; 0.238) \| -0.022(-0.201; 0.157) \| \| T3 \| 0.010(-0.112; 0.132) \| 0.003(-0.163; 0.170) \| 0.010(-0.168; 0.188) \|   ^a^ Associations with PFAS are presented per ln-unit and by quartiles of exposure, as related to ^b^the sex- and gestational age specific Birth Weight Standard Deviation Score (BW-SDS).All analyses were adjusted for maternal weight, parity (3 categories) and cotinine levels. Analyses including both boys and girls were in addition adjusted for sex. |
| --- | --- | --- | --- | --- | --- | --- | --- | --- | --- | --- | --- | --- | --- | --- | --- | --- | --- | --- | --- | --- | --- | --- | --- | --- | --- | --- | --- | --- | --- | --- | --- | --- | --- | --- | --- | --- | --- | --- | --- | --- | --- | --- | --- | --- | --- | --- | --- | --- | --- | --- | --- | --- | --- | --- | --- | --- | --- | --- | --- | --- | --- | --- | --- | --- | --- | --- | --- | --- | --- | --- | --- | --- | --- | --- | --- | --- | --- | --- | --- | --- | --- | --- | --- | --- | --- | --- | --- | --- | --- | --- | --- | --- | --- | --- | --- | --- | --- | --- | --- | --- | --- | --- | --- | --- | --- | --- | --- | --- | --- | --- | --- | --- | --- | --- | --- | --- | --- | --- | --- | --- | --- | --- | --- | --- | --- | --- | --- | --- | --- | --- | --- | --- | --- | --- | --- | --- | --- | --- | --- | --- | --- | --- | --- | --- | --- | --- | --- | --- | --- | --- | --- | --- | --- | --- | --- | --- | --- | --- | --- | --- | --- | --- | --- | --- | --- | --- | --- | --- |
